# Three-dimensional structures of the tracheal systems of *Anopheles sinensis* and *Aedes togoi* pupae

**DOI:** 10.1038/srep44490

**Published:** 2017-03-13

**Authors:** Young-Ran Ha, Eunseop Yeom, Jeongeun Ryu, Sang-Joon Lee

**Affiliations:** 1 Center for Biofluid and Biomimic Research, Pohang University of Science and Technology, Pohang, 790-784, Republic of Korea; 2 School of Mechanical Engineering, Pusan National University, Pusan, 609-735, Republic of Korea; 3 Department of Mechanical Engineering, Pohang University of Science and Technology, Pohang, 790-784, Republic of Korea

## Abstract

Mosquitoes act as a vector for the transmission of disease. The World Health Organization has recommended strict control of mosquito larvae because of their “few, fixed, and findable” features. The respiratory system of mosquito larvae and pupae in the water has a weak point. As aquatic organisms, mosquito larvae and pupae inhale atmosphere oxygen. However, the mosquito pupae have a non-feeding stage, unlike the larvae. Therefore, detailed study on the tracheal system of mosquito pupae is helpful for understanding their survival strategy. In this study, the three-dimensional (3D) structures of the tracheal systems of *Anopheles sinensis* and *Aedes togoi* pupae were comparatively investigated using synchrotron X-ray microscopic computed tomography. The respiratory frequencies of the dorsal trunks were also investigated. Interestingly, the pupae of the two mosquito species possess special tracheal systems of which the morphological and functional features are distinctively different. The respiratory frequency of *Ae. togoi* is higher than that of *An. sinensis*. These differences in the breathing phenomena and 3D structures of the respiratory systems of these two mosquito species provide an insight into the tracheal systems of mosquito pupae.

Mosquitoes undergo four distinct stages in their life cycle: egg, larva, pupa, and adult. Soon after the fourth moult, the larva becomes inactive and remains at the bottom of the water. Then, the metamorphosis into a comma-shaped stage called pupa occurs. Mosquito pupae evolved to adapt for living in the water by using oxygen for respiration^1^. The respiratory system of mosquito pupae, as aquatic insects, has limitations in accessing atmospheric oxygen^1^.

Mosquito pupae remain most of the time on the water surface with the top of its thorax and abdomen hanging down. The head and thorax of a pupa are combined into a cephalothorax. The cephalothorax bears two respiratory trumpets on its dorsal surface^1^,^2^. Pupae do not use the terminal abdominal spiracles for respiration, unlike the larvae^1^. The respiratory trumpets are connected with mesothoracic spiracles coupled with short tracheal trunks and dorsal longitudinal tracheal trunks^1^. These trumpets are moved posteriorly when a pupa dives to minimize the energy spent for overcoming the interfacial tension^3^.

Mosquitoes transfer infectious diseases from person to person. Many control methods, such as source reduction, bio-control, and pesticides have been used to exterminate mosquitoes^4^,^5^. Various pesticides, such as temephos, methoprene, *Bacillus thuringiensis israelensis*, malathion, naled, and pyrethrins, are available for mosquitoes. However, only oils and monomolecular films are suggested for mosquito pupae^5^. These pesticides alter water surface tension by physical means^6^. Development of new methods for controlling mosquito pupae would be helpful. Gaining more detailed knowledge of pupal mosquito tracheal systems may aid our ability to design better physical control systems aimed at interfering with mosquito pupal respiration. The respiratory trumpets and organs of mosquito pupae possess different morphological and functional characteristics depending on mosquito species.

In this study, the 3D morphological features of the respiratory system of *An. sinensis* and *Ae. togoi* pupae were experimentally investigated using synchrotron X-ray microscopic computed tomography (SR-μCT). The tracheal systems were extracted from the 3D reconstructed images of mosquito pupae to compare the anatomical characteristics of the respiratory systems of the two mosquito species. The respiratory frequencies of the tracheal systems of *An. sinensis* and *Ae. togoi* were also compared. These results are helpful to understand the anatomical adaptations and biophysical characteristics of the tracheal systems of *An. sinensis* and *Ae. togoi* pupae.

## Results

### Microscopic images of mosquito pupae

**Figure 1:**
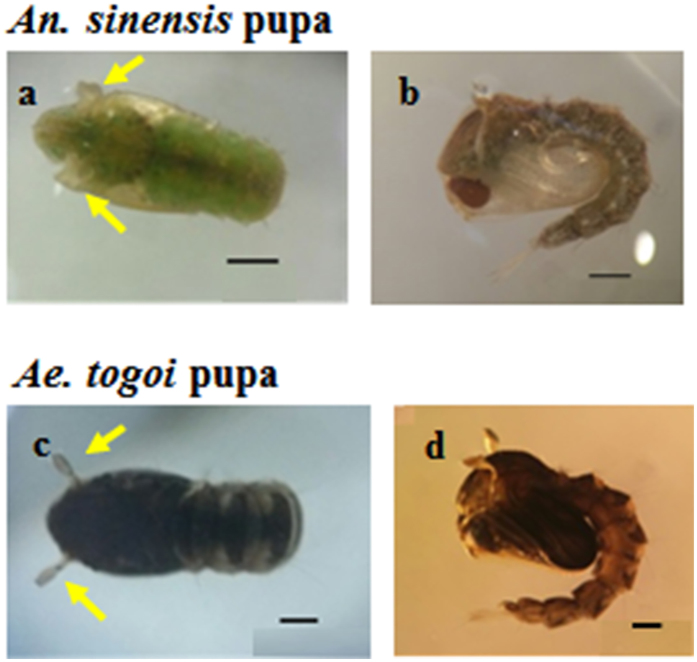
Photographs of *An. sinensis* and *Ae. togoi* pupae. The axial view of *An. sinensis* (**a)** and *Ae. togoi* pupae (**c**). The sagittal view of *An. sinensis* (**b**) and *Ae. togoi* pupae (**d**). The yellow arrows indicate the respiratory trumpets of *An. sinensis* and *Ae. togoi* pupae. Scale bars (**a**–**c**, and **d**) indicate 0.5 mm.

Figure 1 shows typical images of *An. sinensis* and *Ae. togoi* pupae. The axial and side views of *An. sinensis* and *Ae. togoi* pupae are depicted in Fig. 1a,c and b,d, respectively. Pupae possess a comma-shaped configuration. The head and thorax are combined into a cephalothorax. The two large paddles at the end of the abdomen are effective for propulsion^1^. The body lengths of *An. sinensis* and *Ae. togoi* pupae are 5.07 ± 1.79 and 4.56 ± 0.12 mm, respectively (two-tailed *P* value = 0.513, *F* value = 1.17). These statistical data support that the pupae of the two mosquito species do not noticeably differ in body size. Yellow arrows indicate the two respiratory trumpets that bear the cephalothorax of *An. sinensis* and *Ae. togoi* pupae (Fig. 1a and c).

### SEM images of mosquito pupae

**Figure 2:**
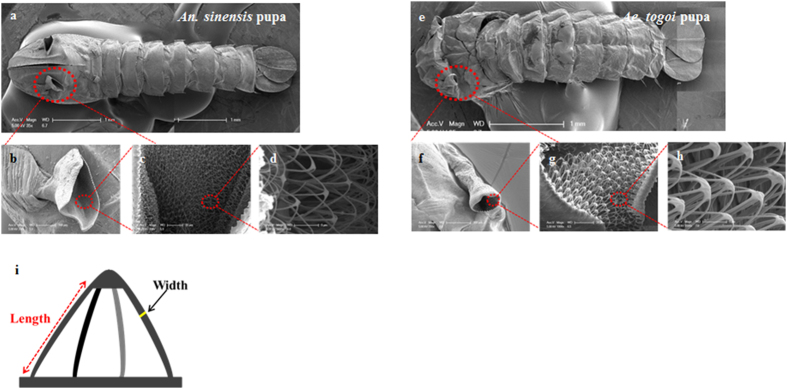
SEM images of the whole body of *An. sinensis* (**a**) and *Ae. togoi* pupae (**e**). The respiratory trumpets of *An. sinensis* (**b**) and *Ae. togoi* pupae (**f**). The meshwork of the respiratory trumpets of *An. sinensis* (**c** and **d**) and *Ae. togoi* pupae (**g** and **h**). (**i**) Schematic sketch showing the structural parameters acquired from the meshwork of the respiratory trumpets: hight and width.

The respiratory trumpets of *An. sinensis* pupa are shorter and broader compared with those of *Ae. togoi* pupa. The trumpets look like a trigonal tube with an inclined cutting line (Fig. 2a and b), whereas *Ae. togoi* pupa possesses funnel-shaped trumpets (Fig. 2e and f). The inner surface of the pinna is covered with cuticular meshwork. The meshwork is composed of dense filamentous materials in the inner surface of the trumpets. The substructures of the meshwork of the two mosquito pupae are notably different. The filament of *An. sinensis* pupa is thinner than that of *Ae. togoi* pupa. *An. sinensis* pupa has usually three to four filaments, whereas *Ae. togoi* pupa has five to six filaments. The widths of the filaments of *An. sinensis* and *Ae. togoi* pupae are 0.38 ± 0.03 and 0.51 ± 0.03 μm (two-tailed *P* value = 0.000473, *F* value = 195.33), respectively. Their lengths are 7.47 ± 1.04 and 10.55 ± 1.00 μm (two-tailed *P* value = 0.00527, *F* value = 45.37), respectively. These results indicate that the filaments in the cuticular meshwork of *Ae. togoi* pupa are more dense than those of *An. sinensis* pupa.

### Nile red staining of mosquito pupae

**Figure 3:**
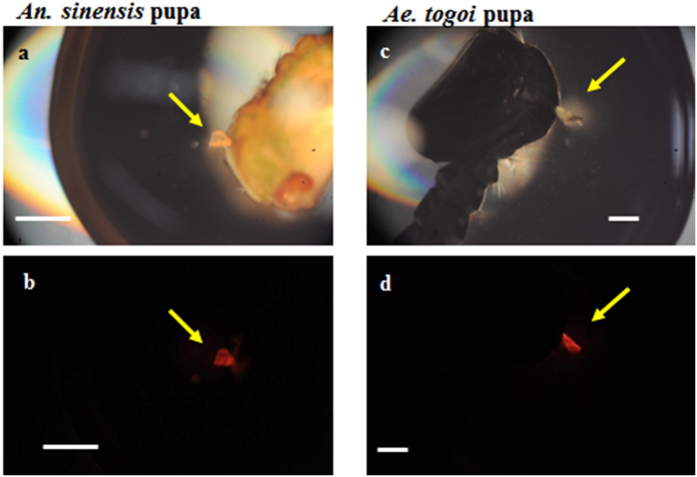
Nile red staining images of the respiratory trumpets of the two mosquito species. Microscopic images of *An. sinensis* (**a**) and *Ae. togoi* pupae (**c**). Fluorescence images of the respiratory trumpets of *An. sinensis* (**b**) and *Ae. togoi* pupae (**d**). Yellow arrows indicate the respiratory trumpets. Scale bars indicate 0.5 mm.

Nile red is a fluorescent dye used as a hydrophobic probe^7^. Nile red has been used to stain neutral lipids^7^. In this study, pupa was stained using Nile red dye to examine the hydrophobic neutral lipids in the respiratory trumpets. Figure 3 shows the fluorescent images of the respiratory trumpets of both *An. sinensis* and *Ae. togoi* pupae. This condition shows that neutral lipids are deposited in the respiratory trumpets of both mosquito pupae.

### 3D structures of larvae and tracheal system

**Figure 4:**
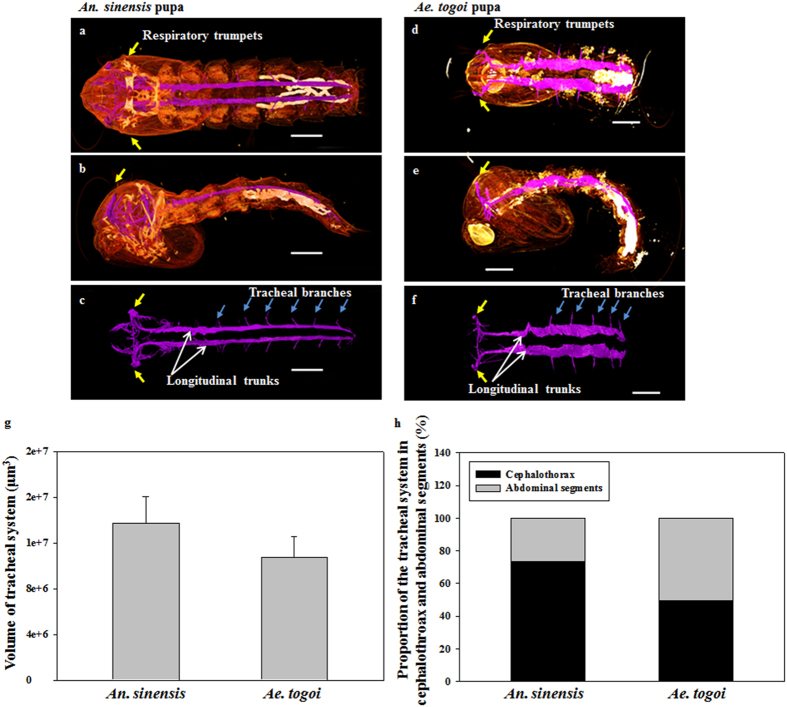
3D morphological structures visualized by SR-μCT. Axial view of the 3D structure of *An. sinensis* (**a**) and *Ae. togoi* (**d**) pupae. Sagittal view of the 3D structure of *An. sinensis* (**b**) and *Ae. togoi* (**e**) pupae. Axial view of the tracheal system of *An. sinensis* (**c**) and *Ae. togoi* (**f**) pupae. (**g**) Comparison of the volume of the tracheal system of both mosquito pupae. (**h**) Proportions of the tracheal system in the cephalothorax and abdomen of both mosquito pupae. Yellow arrows indicate the positions of the respiratory trumpets. Blue arrows represent the tracheal branches. White arrows indicate the dorsal longitudinal trunks. Scale bars indicate 0.5 mm.

Figure 4 shows the axial and sagittal images of the tracheal system of both mosquito pupae visualized using SR-μCT. The morphological structures of the tracheal system of both mosquito species were comparatively analyzed to evaluate the anatomical features of their respiratory systems. The tracheal system of a mosquito pupa is composed of longitudinal trunks and tracheal branches. Yellow arrows in Fig. 4 indicate the positions of the respiratory trumpets. The pupae of the two mosquito species exhibit noticeable morphological differences in the tracheal system, especially in the cephalothorax and abdominal segments. In the cephalothorax part, the tracheal system of *An. sinensis* pupa is more developed compared with that of *Ae. togoi*. The anatomical width of the dorsal trunks of *Ae. togoi* pupa is wider than that of *An. sinensis* (Fig. 4a and d). The tracheal branches of the two different mosquito pupae are depicted in Fig. 4c and f. These results show that the tracheal systems of *An. sinensis* and *Ae. togoi* pupae have distinctly different morphological structures.

The volumes of the tracheal system ranged from the cephalothorax to the end of the abdominal segments of the two mosquito species were compared. Three to five pupa samples of similar sizes were statistically averaged to estimate the volumes of their tracheal systems. The total volumes of the tracheal system of *An. sinensis* and *Ae. togoi* are 13.74 ± 2.32 × 10^−3^ and 10.73 ± 1.81 × 10^−3^ mm^3^, respectively (Fig. 4g). The tracheal volumes of *An. sinensis* and *Ae. togoi* pupae are not statistically different (two-tailed *P* value = 0.152). The volume proportions of the tracheal system in the cephalothorax and abdominal segments to the total volume are approximately 73.30% and 26.69% for *An. sinensis.* The corresponding values of *Ae. togoi* are 49.36% and 50.63% for the cephalothorax and abdominal segments, respectively (Fig. 4h).

### Cross-sectional images of mosquito pupae

**Figure 5:**
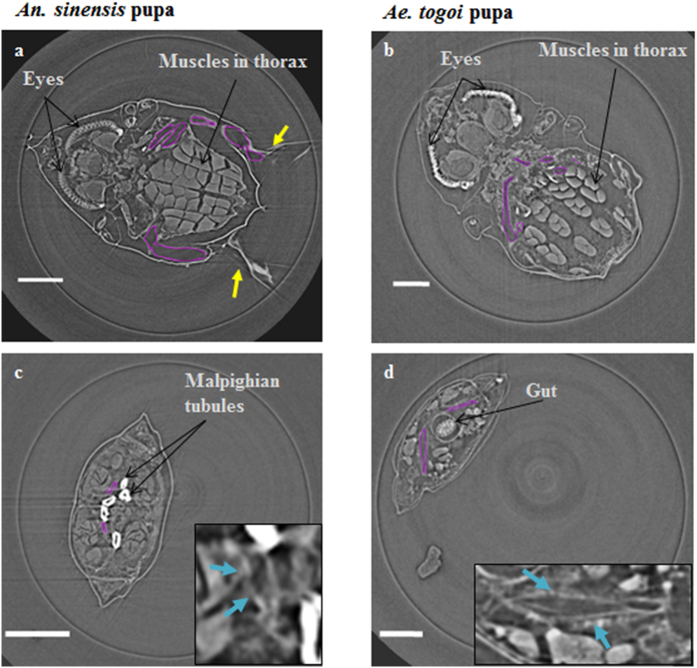
Cross-sectional images of the tracheal system visualized using SR-μCT. Cross-sectional images of the tracheal system in the cephalothorax of *An. sinensis* (**a**) and *Ae. togoi* (**b**) pupae. Cross-sectional images of the tracheal system in the abdomen of *An. sinensis* (**c**) and *Ae. togoi* (**d**) pupae. Insets show the magnified right side of the tracheal system (blue arrows). The purple lines represent the tracheal system inside of the cephalothorax. Yellow arrows indicate the positions of the respiratory trumpets. Scale bars indicate 0.25 mm.

Figure 5a and b show the cross-sectional views of the cephalothorax part of *An. sinensis* and *Ae. togoi* mosquito pupae, respectively. The concealed head and thorax are clearly observed in the cross-sectional images. Figure 5c and d indicate the tracheal organ in the abdominal segments of *An. sinensis* and *Ae. togoi* mosquito pupae, respectively. The widths of the DLT in the abdominal segments of *An. sinensis* and *Ae. togoi* pupae are 0.056 ± 0.012 and 0.167 ± 0.0.044 mm (two-tailed *P* value = 0.0000237, *F* value = 50.99), respectively. Thus, the tracheal organ in the abdominal segments of *Ae. togoi* pupa is much larger, compared with that of *An. sinensis*.

### Analysis of breathing behavior

**Figure 6:**
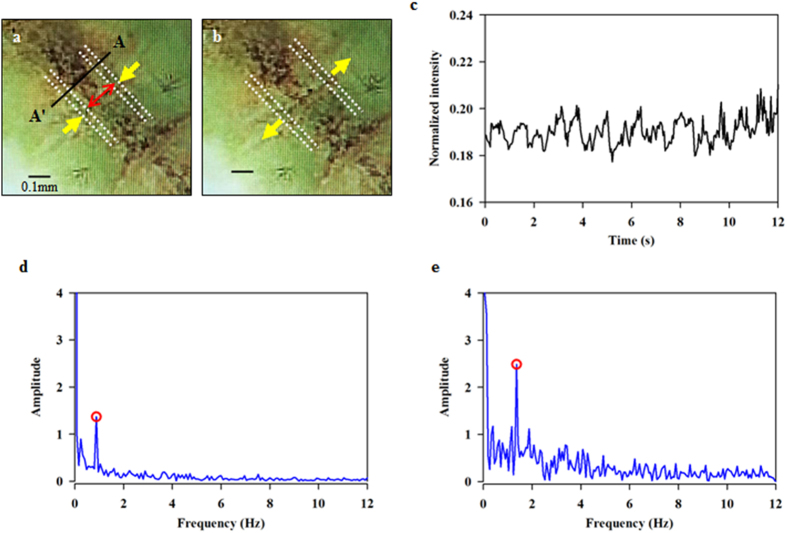
Variations of respiratory frequency of the tracheal systems of *An. sinensis* and *Ae. togoi* pupae. Yellow arrows indicate the dorsal trunks in the cephalothorax of mosquito pupae at the contraction (**a**) and expansion phase (**b**). Cross-sectional line AA′, from which variations in the inner diameters and frequency profiles of the two dorsal trunks are extracted. (**c**) Normalized intensity variation shows the respiratory motion of the dorsal trunks of *Ae. togoi* pupae. Peristaltic frequency variations in the dorsal trunks and the peak frequencies of *An. sinensis* (**d**) and *Ae. togoi* (**f**) pupae. Yellow arrows indicate the moving directions of the dorsal trunks. Red arrows denote the inner diameter of the dorsal trunks. Scale bar indicates 0.1 mm.

We estimated the respiratory frequencies in the tracheal system of the two mosquito pupae from the dynamic variations of the dorsal trunks. Consecutive images of the dorsal longitudinal trunks exhibit peristaltic motions (Fig. 6a and b). The dorsal trunks show phasic variations of contraction and relaxation during respiratory process. The phasic variations of the inner diameter of the dorsal trunks were extracted from the cross-sectional line AA′ marked in Fig. 6a. A typical normalized phasic intensity variation is shown in Fig. 6c. The peak frequency of the dorsal trunks during their respiratory motions was analyzed using spectral analysis by applying fast Fourier transform to the temporal variations of the inner diameter of the trunks. The power spectral density signals of the dorsal trunks are depicted in Fig. 6d and e. The peak respiratory frequencies of *An. sinensis* and *Ae. togoi* pupae are 0.88 ± 0.09 and 1.36 ± 0.38 Hz (two-tailed *P* value = 0.0473, *F* value = 9.67), respectively.

## Discussion

Pupae belong to the non-feeding stage and rely on the food stored in the larval stage^8^. Their resting tendency without any disturbance is obviously aimed at conserving energy^9^. Therefore, they should consider energy balance between oxygen consumption and energy expenditure to avoid any unexpected situations, such as predation^2^. In addition, the tracheal system of insect is commonly the major conduit used for spreading virus to the host. The rapid spread of virus infection through the tracheal system is facilitated by the epidermal lymph system^10^. Therefore, the respiratory system of pupae exhibits a strong influence on their survival.

Pupae utilize gas in the ventral air space to be buoyant. Submerging pupae become negatively buoyant. They recover positive buoyancy by a brief contact with atmospheric air. This shift in buoyancy of pupae could be affected by changes in gas pressure and/or volume in the tracheal system^11^.

In a previous study, the spiracular opening was observed to be encircled by a ring of hydrofuge cuticle, which resists entry of water^1^. By contrast, a great part of the outer surface of the respiratory trumpets exhibits hydrophilic property^1^. The differences in the respiratory organs of mosquito larvae may have noticeable influence on the morphology and functions of mosquito pupae. In this study, the shapes of respiratory trumpets and the meshworks of the two different mosquito pupae are clearly different (Fig. 2). The meshwork is a hydrofuge, which prevents entry of water into the respiratory system^1^. In a previous study, several plants or animals possess complicated structural surfaces to hold air films under water for a very long period of time^12^. Such air films have been used in various applications, such as long-term respiration^12^. The meshwork of the respiratory trumpets of mosquito pupae is composed of several filaments, as shown in Fig. 2. The terminal ends of the filaments are connected, forming an eggbeater-shaped structure which is used to trap a thin air layer^12^. The eggbeater-shaped structure effectively supports the air–water interface in mechanical point of view^12^. Therefore, certain energy is required for the filaments of the eggbeater-shaped structure to penetrate the water surface^12^. The meshwork is divided into several filaments, which effectively stabilize the air-water interface^12^. The difference in length and width of the filaments of the respiratory trumpets may lead to dissimilar energy efficiencies in living on the water surface, while preventing submersion and wetting. In this study, we compared penetration of the respiratory trumpets through an oil-treated water surface (Fig. S1). Under the same condition, the respiratory trumpets of *Ae. togoi* pupa can pierce the oil-treated water surface, whereas those of *An. sinensis* pupa can not penetrate the surface. This result implies that the more dense form of filaments in the respiratory trumpets of *Ae. togoi* pupa may provide them with an advantage during respiration in water that is covered with oil. However, a further detailed study is required to explain the effects of different structures of the filaments on the stabilization of air–water interface.

In this study, the movement of dorsal longitudinal trunks was analyzed during respiratory process (Fig. 6). Since the tracheal system of live mosquito pupae is flexible, it is not totally dependent on diffusion for oxygen delivery. A previous study reported that the tracheal system of Drosophila embryos has a chitin-matrix which helps to expand the tracheal chitin cylinder^13^,^14^. The serosal cuticle of a mosquito contains chitin which resists desiccation^15^. However, the molecular nature of the tracheal system and the respiratory trumpets of mosquito pupae are still unknown. Future study on the existence of chitin-matrix that lines the apical cell surfaces of the trumpets would be helpful for understanding the tracheal function.

In conclusion, the breathing phenomena and 3D morphological structures of the different respiratory systems of larvae of the two mosquito species were investigated. These results could provide in-depth understanding of tracheal systems of the *An. sinensis* and *Ae. togoi* pupae.

## Methods

### Mosquito rearing and sample preparation

Following the established rearing procedures^16^, mosquitoes (*An. sinensis* s.l. and *Ae. togoi* Theobald 1907) were fed a 10% sugar solution at 27 °C with 80% humidity in a 16 h:8 h light/dark cycle. Larvae were fed a slurry of ground fish food and baker’s yeast. After pupation, the mosquito pupae were transferred to a cage and provided a 10% sucrose-soaked cotton rod.

### Scanning electron microscopy

Scanning electron microscopy (SEM) was employed to illustrate the morphological configurations of the test samples. Mosquito specimens were fixed with ethanol and submerged in hexamethyldisilazane or prepared by air drying. The samples were then Ag-coated using a coater (Quorum Technology, SC7640 mode, East Sussex, United Kingdom) and examined by a field emission SEM (XL30S FEG, Philips Electron Optics B.V., the Netherlands) connected to an EDXS system at an acceleration voltage of 5 kV.

### Staining pupae with Nile red

Nile red (Sigma-Aldrich, St. Louis, MO, USA) was dissolved in acetone (500 μg/ml) and stored at 4 °C under darkness to prepare the stock solution for Nile red staining. Approximately 10 μl of stock solution was added to 10 ml of 75% glycerol. The Nile red staining solution was applied to pupae for 5–10 min to stain the respiratory trumpets of pupae^7^. After staining, pupae were washed with fresh water to avoid any non-specific binding of Nile dyes in their body. The stained pupae were examined using a fluorescent microscope (Nikon, Tokyo, Japan).

### Synchrotron X-ray microscopic computed tomography (SR-μCT)

All SR-μCT experiments were performed at the Biomedical Imaging beamline of the Pohang Light Source-II at Pohang, Korea. This beamline was newly constructed and opened for general users in September 2013. A white beam emitted from the multi-pole wiggler source of the 3 GeV storage ring was filtered with a graphite of 1 mm thickness after passing through a double multilayer monochromator, providing photon energy in the range from 10 keV to 50 keV. The optimal monochromatic X-ray energy for the present tomographic scanning experiments was experimentally determined to be approximately 24 keV. The detector was positioned at 10–20 cm downstream from the test sample for capturing phase-contrast images, depending on experimental conditions. The monochromatic X-ray image transmitted through the test sample was recorded by an imaging detector (Andor Zyla). The field of view was 2560 × 2160 pixels along the horizontal and vertical directions. An X-ray image was converted into a visible image on the YAG:Ce (30 μm thick) scintillation crystal adhered at the image detector. All samples of mosquito pupae immersed in ethanol were placed at the tip of a heat-sealed pipette. The pipette was made of polypropylene that has a comparatively low X-ray absorption coefficient^17^. Parafilm was used to prevent evaporation of alcohol. Each test sample was placed on a rotating sample stage. 2D tomographic slice images of the sample were captured by rotating the sample stage from 0° to 180° at 0.5° interval. The spatial resolution was 1.625 μm/pixel, which was based on the pixel size of this camera with a 4x objective lens. The 3D morphological structure of each mosquito sample was reconstructed from the captured tomographic slice images using the Octopus image-processing program. A filtered back-projection algorithm was applied to the projection images to obtain the 3D volumetric image of the sample. Cross-sectional images were stacked. These images were used to provide a 3D morphological structure using the Amira^®^ 5.3.3 image-analysis software (Visualization Sciences Group, Burlington, MA, USA). The tracheal organs were quantitatively evaluated by clustering the pixels in the salient image regions using Amira^®^ 5.3.3 image-analysis software.

### Analysis of respiratory frequency

Samples were mounted in a stereo microscope (Stemi 2000-C, Zeiss, Germany) equipped with an objective lens of 1.6× magnification. A high-speed CMOS camera (FASTCAM SA 1.1, Photron Ltd., San Diego, USA) was used to acquire consecutive images at a frame rate of 125 fps. Inner diarmeters of the dorsal trunks of the tested pupae were evaluated using a digital image-processing technique based on the Otsu’s thresholding method^18^. The temporal variations of the inner diameters of the pupae were analyzed using FFT (Fast Fourier transform)^19^,^20^ to evaluate the respiratory frequencies of the two mosquito species.

### Statistical analysis

Statistical analysis was performed using Sigma Plot 13.0 (Jandel Scientific, Corte Madera, CA, USA).

## Additional Information

**How to cite this article**: Ha, Y.-R. *et al*. Three-dimensional structures of the tracheal systems of *Anopheles sinensis* and *Aedes togoi* pupae. *Sci. Rep.* **7**, 44490; doi: 10.1038/srep44490 (2017).

**Publisher's note:** Springer Nature remains neutral with regard to jurisdictional claims in published maps and institutional affiliations.

## Supplementary Material

Supplementary Video 1

Supplementary Video 2

Supplementary Video 3

Supplementary Information

## References

[b1] Clements A. N. *The Biology of Mosquitoes: Volume 1: Development, Nutrition and Reproduction*. XV–XVI, 119–120 (CABI, 1992).

[b2] Awasthi A. K., Wu C. H. & Hwang J. S. Diving as an Anti-Predator Behavior in Mosquito Pupae. Zool Stud. 51, 1225–1234 (2012).

[b3] Reiter P. The action of lecithin monolayers on mosquitoes. II. Action on the respiratory structures. Ann Trop Med Parasitol. 72, 169–176 (1978). 27147 10.1080/00034983.1978.11719300

[b4] Chang M. S. . Challenges and future perspective for dengue vector control in the Western Pacific Region. Western Pac Surveill Response J. 2, 9–16 (2011). 23908883 10.5365/WPSAR.2010.1.1.012 PMC3730960

[b5] Rose R. I. Pesticides and public health: integrated methods of mosquito management. Emerg Infect Dis. 7, 17–23 (2001). 11266290 10.3201/eid0701.010103 PMC2631680

[b6] Nayar J. K. & Ali A. A review of monomolecular surface films as larvicides and pupicides of mosquitoes. J Vector Ecol. 28, 190–199 (2003). 14714668

[b7] Fowler S. D. & Greenspan P. Application of Nile red, a fluorescent hydrophobic probe, for the detection of neutral lipid deposits in tissue sections: comparison with oil red O. J Histochem Cytochem. 33, 833–836 (1985). 4020099 10.1177/33.8.4020099

[b8] Lucas E. A. Jr. & Romoser W. S. The energetic costs of diving in Aedes aegypti and Aedes albopictus pupae. J Am Mosq Control Assoc. 17, 56–60 (2001). 11345420

[b9] Romoser W. S. & Lucas E. A. Buoyancy and diving behavior in mosquito pupae. J Am Mosq Control Assoc. 15, 194–199 (1999). 10412114

[b10] Engelhard E. K., Kam-Morgan L. N., Washburn J. O. & Volkman L. E. The insect tracheal system: a conduit for the systemic spread of Autographa californica M nuclear polyhedrosis virus. Proc Natl Acad Sci USA. 91, 3224–3227 (1994). 8159729 10.1073/pnas.91.8.3224 PMC43548

[b11] Romoser W. S. Buoyancy and ventilation in Aedes aegypti (L.) pupae (Diptera: Culicidae). J Med Entomol. 12, 547–550 (1975). 1223305 10.1093/jmedent/12.5.547

[b12] Barthlott W. . The salvinia paradox: superhydrophobic surfaces with hydrophilic pins for air retention under water. Adv Mater. 22, 2325–2328 (2010). 20432410 10.1002/adma.200904411

[b13] Luschnig S., Batz T., Armbruster K. & Krasnow M. A. serpentine and vermiform encode matrix proteins with chitin binding and deacetylation domains that limit tracheal tube length in Drosophila. Curr Biol. 16, 186–194 (2006). 16431371 10.1016/j.cub.2005.11.072

[b14] Moussian B. . Drosophila Knickkopf and Retroactive are needed for epithelial tube growth and cuticle differentiation through their specific requirement for chitin filament organization. Development. 133, 163–171 (2006). 16339194 10.1242/dev.02177

[b15] Farnesi L. C., Menna-Barreto R. F., Martins A. J., Valle D. & Rezende G. L. Physical features and chitin content of eggs from the mosquito vectors Aedes aegypti, Anopheles aquasalis and Culex quinquefasciatus: Connection with distinct levels of resistance to desiccation. J Insect Physiol. 83, 43–52 (2015). 26514070 10.1016/j.jinsphys.2015.10.006

[b16] Kim B. H., Kim H. K. & Lee S. J. Experimental analysis of the blood-sucking mechanism of female mosquitoes. J. Exp. Biol. 214, 1163–1169 (2011). 21389202 10.1242/jeb.048793

[b17] Metscher B. D. MicroCT for comparative morphology: simple staining methods allow high-contrast 3D imaging of diverse non-mineralized animal tissues. BMC physiol. 9, 11 (2009). 19545439 10.1186/1472-6793-9-11 PMC2717911

[b18] Otsu N. A Threshold Selection Method from Gray-Level Histograms. IEEE-TSMC. 9, 62–66 (1979).

[b19] Tonar Z., Němeček S., Holota R., Kočová J., Třeška V., Moláček J., Kohoutek T. & Hadravská S. Microscopic image analysis of elastin network in samples of normal, athersclerotic and aneurysmatic abdominal aorta and its biomechanical implications. J. Appl. Biomed. 1, 149–159 (2003).

[b20] Taylor S. E., Cao T., Talauliker P. M. & Lifshitz J. Objective Morphological Quantification of Microscopic Images Using a Fast Fourier Transform (FFT) Analysis. Curr Protoc Essent Lab Tech. 95, 9 5 1–9 5 12 (2013). 27134700 10.1002/9780470089941.et0905s07 PMC4849894

