# Supplementary material for: Three-dimensional structures of the tracheal systems of Anopheles sinensis and Aedes togoi pupae
Source: Sci Rep. 2017 Mar 13;7:44490. doi: 10.1038/srep44490 (PMC5347383; doi:10.1038/srep44490)
Supplement: Supplementary Information [file srep44490-s4.pdf]

Three-dimensional structures of the tracheal systems of *Anopheles sinensis* and *Aedes togoi* pupae

Young-Ran Ha[1], Eunseop Yeom[2], Jeongeun Ryu[1] and Sang-Joon Lee[1,3]*

[1]Center for Biofluid and Biomimic Research, Pohang University of Science and Technology, Pohang, 790-784, Republic of Korea

[2] School of Mechanical Engineering, Pusan National University, Pusan, 609-735, Republic of Korea

[3]Department of Mechanical Engineering, Pohang University of Science and Technology, Pohang, 790-784, Republic of Korea

[*]Corresponding author: **Prof. Sang Joon Lee**

Center for Biofluid and Biomimic Research, Department of Mechanical Engineering, Pohang University of Science and Technology, San 31, Hyojadong, Namgu, Pohang, Kyungbook, 790-784, Republic of Korea

Tel.: +82-54-279-2169, Fax: +82-54-279-3199, E-mail: sjlee@postech.ac.kr

Supplementary materials and methods

Olive oil (Sigma-Aldrich, St. Louis, MO, USA) was used to test compare respiration of *An. sinensis* and *Ae .togoi* pupae. 300 ul of olive oil was applied to 700ml of water in PYREX^{TM} Spot plate (Fisher Scientific, Pittsburgh, PA, catalog 722085). The movements of the respiratory trumpets of both mosquito pupae were observed using a stereoscopic microscope (Stemi DV4, Carl Zeiss, Germany).

Supplementary results

Supplementary Video 1. This video shows the cross-sectional view of –X-ray image stack from the cephalothorax to the 3rd abdomen of *An. sinensis* pupa. The puple line indicates the tracheal system.

Supplementary Video 2. This video shows the cross-sectional view of –X-ray image stack from the cephalothorax to the abdomen of *Ae. togoi* pupa. The puple line indicates the tracheal system.

Supplementary Video 3. This video shows the movement of tracheal organ of *Ae. togoi* pupa. This movie exhibits the peristaltic motions of dorsal longitudinal trunks.

Supplementary Figure 1. The effect of surface tension of water are investigated using olive oil. Under the same condition, the respiratory trumpets of *Ae. togoi* pupa penetrates the water surface mixed with olive oil (Fig. S1a). However, the respiratory trumpets of *An. sinensis* pupa can not penetrate the oil-treated water surface (Fig. S1b).

Supplementary figure

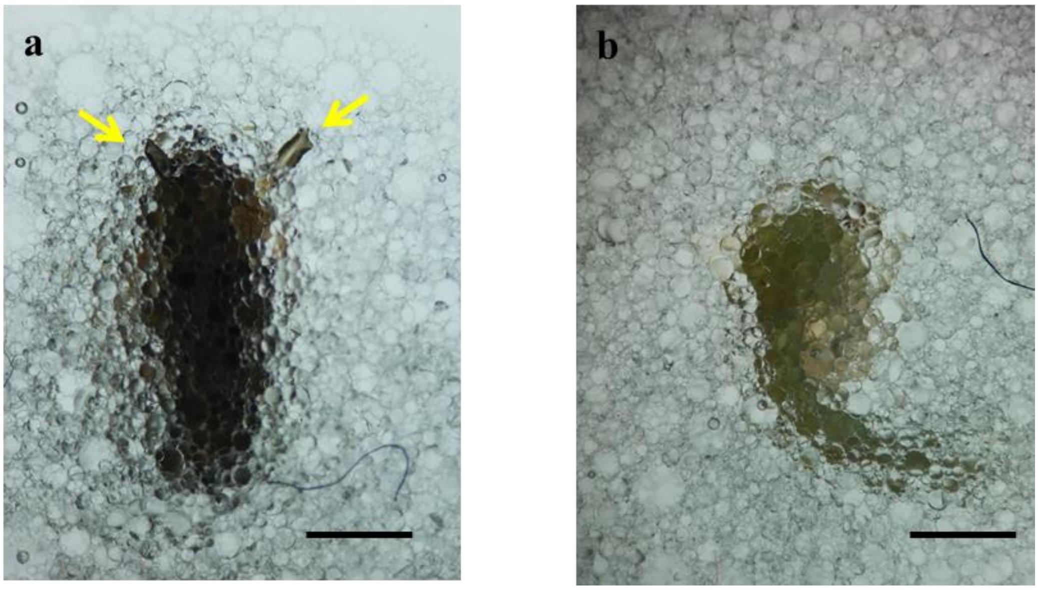

Supplementary video legends

Supplementary video. V1. The cross-sectional views of the cephalothorax to the 3rd abdomen of *An. sinensis* pupa.

Supplementary video. V2. The cross-sectional views of the cephalothorax to the abdomen of *Ae. togoi* pupa.

Supplementary video. V3. The movement of tracheal organ of *Ae. togoi* pupa.

Supplementary Figure S1. Phtographs of *Ae. togoi* and *An. sinensis* Pupae at the oil-treated water surface. Axial image of *Ae. togoi* (a) and *An. sinensis* (b) pupae. The yellow arrows indicate the respiratory trumpets. Scale bars represent 1 mm.
